# Neurodevelopmental considerations for transcranial magnetic stimulation trials in youth

**DOI:** 10.1038/s41386-025-02225-w

**Published:** 2025-09-08

**Authors:** Christine A. Conelea, Alana Lieske

**Affiliations:** Department of Psychiatry & Behavioral Sciences, University of Minnesota, Minneapolis, MN USA

**Keywords:** Outcomes research, Development of the nervous system

## Abstract

Repetitive transcranial magnetic stimulation (rTMS) is an emerging treatment for neuropsychiatric disorders that shows initial efficacy, safety, and tolerability in adolescents with treatment-resistant depression. As research expands to clinical trials testing rTMS in youth with other diagnoses and at younger ages, it is important to consider how neurodevelopmental factors might moderate or mediate rTMS effects and factor this into clinical trial design. In the current paper, we review how key domains of neurodevelopment may interact with rTMS, including neuroanatomy, neural circuit network topography, neuroplasticity, hormones, state-dependent effects, and psychosocial development. We also highlight approaches that can be taken in trials to enhance developmental adaptations of rTMS while also balancing ethical considerations, safety, feasibility, and tolerability. Future directions for research as we move into the “efficacy era” of pediatric rTMS research are discussed.

## Introduction

Over the past few decades, developmental neuroscience research has revealed the prominent role of functional neurocircuit alterations in the onset and maintenance of child and adolescent psychiatric disorders. Advances in neuroimaging have enabled methods to reliably identify and map these networks, including at the individual-level [1]. Researchers are actively studying how particular network-level disruptions may contribute to aberrant cognitive processes and psychiatric symptoms. This includes efforts to understand how particular networks give rise to particular symptoms, in whom this occurs, and how this unfolds over different stages of illness. Increased knowledge about network functionality in psychiatric disorders will open up opportunities to translate findings into circuit-based therapeutics.

A promising tool for non-invasive, circuit-based intervention in youth is repetitive transcranial magnetic stimulation (rTMS). Research in adults demonstrates that particular rTMS protocols can durably modulate brain networks and improve psychiatric functioning. For example, rTMS is FDA-approved for treatment-resistant major depressive disorder (TRD) and obsessive compulsive disorder (OCD) in adults. Pediatric rTMS intervention research is comparatively lacking, although rTMS was recently cleared by the FDA for TRD in adolescents aged 15 years and older [2]. Safety and tolerability have consistently been demonstrated in preliminary studies of youth with a variety of psychiatric and neurological disorders, and side-effects tend to be transient and mild [3].

During TMS, electrical current is pulsed through a small magnetic coil positioned over a targeted area on the scalp, generating a brief, high-intensity magnetic field. This induces an electrical field typically reaching 1–3 cm into the cortex that can depolarize neurons to the point of triggering action potentials [4]. Magnetic stimulation of the cortex was introduced by Barker et al. in 1985 [5] as an alternative to transcranial electric stimulation for assessment of the corticospinal tract, marking 40 years of research using this technology in adults. Applying TMS in single or paired pulses allows for measurement of corticomotor and cortico-cortical responses which can be captured using electromyography (EMG) and electroencephalography (EEG), respectively.

Repetitive TMS (rTMS) uses trains of pulses to induce cortical effects that outlast the duration of stimulation, enabling various forms of neuroplasticity. TMS devices can be operated within a large parameter space, including stimulation intensity, frequency, and duration; cortical target selection; coil positioning and orientation; and the number of sessions and their spacing. Coil placement can be guided by scalp-based measurements or neuronavigation equipment to locate group averaged or individually defined MRI/fMRI-guided coordinates. The terms “targeting” and “coil placement” are synonymous and refer to the methods used to select the TMS coil location and orientation during stimulation. Neuromodulation of the targeted circuit is commonly referred to as “circuit engagement.” rTMS outcomes reflect the cumulative effects of all of these parameters and how they interact with an individual’s brain structure, function, and state [6]. For more in-depth and comprehensive descriptions of TMS methods and mechanisms, see Klomjai et al. [7] and Hallett [8].

As the field works to extend rTMS interventions to children and adolescents, a critical consideration is how developmental factors might moderate or mediate rTMS effects. Commonly used terms to stratify development reflect chronological age and the “typical” or “normative” biological, cognitive, and psychosocial milestones associated with each (i.e., infancy ≈0–2 years, early childhood ≈2–6 years, middle childhood ≈6–11 or 12 years, adolescence ≈11–18 or up to 25 years, emerging adulthood ≈18–25 years; [9]). Herein, we use the terms “pediatric” and “youth” to encompass all groups younger than 18 years, and “childhood” and “adolescence” to distinguish between youth that are on either side of puberty. However, we use these categories with three important caveats. First, development is a multi-staged, non-linear, dynamic, and heterogeneous experience that encompasses change across many interactive domains (e.g., biological, cognitive, social, emotional, academic/occupational). Trajectories of developmental changes are influenced by internal (e.g., genetics) and external factors (e.g., experiences, environmental context) and their bidirectional relationships. Second, chronological age can be discordant from developmental stage, particularly in those with neurodevelopmental disorders. Finally, the phenotypic expression of psychiatric disorders can also vary developmentally, likely reflective of both neurally- and contextually-mediated factors.

We have long known that youth are not just “little adults.” For rTMS intervention research, this means we must be cautious about “copy+pasting” adult rTMS protocols for clinical trials in youth. Here, we highlight several key considerations for developmentally-informed rTMS intervention design and trial implementation (summarized in Table 1).

**Table 1:** Summary of developmental considerations, related challenges, and possible solutions and future directions for rTMS intervention research in children and adolescents.

Developmental consideration	Related challenges	Current possible solutions	Future directions
Neuroanatomy	• Dynamic change in cortical thickness, myelination, synaptic pruning, white and gray matter volume • Higher resting motor threshold in youth vs. adults • Unreliable resting motor threshold • Higher variability, field strength, and distribution of the TMS-induced electric field in youth vs. adults	• Individual-level modeling of the TMS-induced electric field, with a focus on scalability to clinical settings • Adjusting rTMS intensity • Longitudinal re-assessment of resting motor threshold	• TMS mechanisms research to include developmental focus • Research focused on interactions between rTMS targets, stimulation parameters, and cortical maturation
Neural network topography	• Shift in network functional connectivity (from short-range to long range) and border refinement with age • Inter-individual heterogeneity network topography	• Network-based targeting focused on aberrant or compensatory networks driving specific domains of functioning • Measure network connectivity in clinical trials • Individualized, network-based rTMS targeting, with a focus on scalability to clinical settings	• Examine the extent to which clinical responses differ by rTMS targeting strategy • Simulation studies using developmental neuroimaging data • Compare inputs to individual FEM models, e.g., fMRI, DTI • Assess interactions between rTMS, developmental stage, and network connectivity
Neuroplasticity	• Heightened neuroplasticity in different areas of the brain during different critical windows of development • Metaplasticity and homeostatic mechanisms	• Longitudinal assessment to understand durability of effects	• Dose-response studies to clarify the neuroplastic effects of rTMS protocols and brain region x development interactions • More rigorous measurement of rTMS effects on the brain outside of the motor cortex
Neurotransmitters	• Change in E/I balance with age • E/I imbalance causing altered rTMS risk profile	• Inclusion of glutamate/GABA measurement in rTMS trials • Closer monitoring of side effects that could be exacerbated by E/I imbalance	• Measurement of regional glutamate/GABA longitudinally in children with clinical indications possibly amenable to rTMS
Hormones	• Altered cortical excitability and neuroplasticity due to fluctuating sex hormones	• Measurement of pubertal stage and exogenous and endogenous hormones in rTMS trials	• Studies to clarify potentially moderating effects of hormones and pubertal stage on cortical excitability, rTMS-induced neuroplasticity, and rTMS outcomes
State-dependent effects	• Behavioral activities and brain state around the time of rTMS can moderate effects • Influential factors are more unstable in youth (e.g., sleep, diet, substance intake, affective state) • Self- and parent-report limitations • Interactions between TMS pulses and neuronal oscillations	• Careful measurement of factors likely to influence brain state to enable quantitative examination of possible moderating effects • Obtain input from multiple informants • Document and report strategies used to enable tolerability during rTMS administration • Leverage state-dependency to inform multimodal treatment design (e.g., pairing with behavioral therapy)	• Studies to examine the feasibility and relative efficacy of EEG-TMS in youth • Studies testing augmentation of rTMS with different cognitive and behavioral therapies
Psychosocial development	• Range of psychosocial capacities across youth can impact rTMS feasibility, including variable reliance on caregivers • Adverse childhood experiences	• Inclusion of professionals with expertise in child development and the target population on the research team • Patient-centered approaches, including patient advisors • Need for researchers to publish protocols and qualitative studies to share “lessons learned” about developmental tailoring of rTMS procedures	• Studies to examine how trauma-related neural changes may interact with rTMS
Safety	• Higher resting motor thresholds can increase risk of seizure • Limitations in youth self-report of baseline status, adverse event experiences, and risk factors • High baseline rates of symptoms that can be common rTMS adverse events, such as headache	• Follow best-practice rTMS safety guidelines, and ensure pediatric experts are included in future guideline updates • Use of EMGs on peripheral muscles for resting motor threshold determination and rTMS parameter adjustments for higher-risk youth • Careful establishment of baseline symptoms and ongoing inquiry and observation to monitor for risk factors	• Structured measures for adverse event monitoring that are developmentally sensitive • Standardized reporting expectations should be adopted by the field
Tolerability	• Heightened sensory sensitivity and lower ability to tolerate discomfort in children vs. adults • More frontal targets can cause nerve/muscle pain	• Study-specific protocols to guide rTMS adjustments to address tolerability and reporting of adjustment frequency and type in published reports • Efforts to reduce uncertainty and overall comfort	• Efforts to include child life specialists in rTMS protocol design • Device or protocol modifications to enhance tolerability in youth, possibly with diagnosis-specific adaptations
Feasibility	• Logistical, organizational, and financial burdens on youth and caregivers • Ethnoracial disparities in study participation	• Engage with patient stakeholders at the stage of trial design to proactively address barriers to participation • Focus on strategies that will be scalable to the clinic	• Dose-response measurement to identify minimal effective doses • Testing of accelerated rTMS protocols
Ethics	• Diminished autonomy and capacity to consent • Parent/caregiver influence • Ethical use of rTMS for treatment vs. “enhancement”	• Facilitate autonomous decision making with developmentally-appropriate education about rTMS procedures and risks • Contextualize rTMS within available treatment options	• Expert consensus statements around the ethical use of neuromodulation for enhancement in children are needed

### Neuroanatomy

The spread and depth of the TMS-induced electrical field is determined by brain anatomy and the physical properties of tissue underneath the coil, including amounts of skin, skull, and cerebral spinal fluid (CSF) [10]. A limited number of these factors are relatively static across child development. Total ventricular CSF volume is consistent between ages 2–30 years [11], and gyral geometry is relatively stable by age 1 year (although gyral refinement in prefrontal regions occurs between ages 6–16 years, and disorder-specific processes can further alter gyrification [12]).

Other aspects of brain structure are much more developmentally dynamic. Myelination, synaptic pruning, white and gray matter volume, and cortical thickness change at non-linear rates that vary regionally across the brain, with slightly higher within-subject variability in younger children [11, 13–15]. The earliest cortical maturation occurs in lower-order primary sensory and motor areas, which reach peak maturation around ages 4–7 years, followed by upper and lower parietal lobe areas involved in language, speech, spatial orientation, and attention (~6–12 years). Development is more protracted higher-order association areas, including prefrontal, orbitofrontal, and fronto-temporal association areas involved in executive function, attention, and motor coordination (~10–25 years), as well as limbic-associated cortices involved in emotion regulation, motivation, and social cognition (e.g., orbitofrontal cortex ~11–13 years, insula ~10–13 years, anterior cingulate cortex ~10–12 years). Interestingly, the frontopolar cortex–an rTMS target of interest for OCD, depression/TRD, substance use disorders, and PTSD–is involved in taste and smell processing and matures very early (~2 years; [11]). Notably, cortical thickness, brain volume, and surface area have shown significant differences in those with psychiatric disorders vs. non-affected controls, with distinct profiles clustering around neurodegenerative, mood and anxiety, and neurodevelopmental disorders. These differences are most prominent in adolescence and late adulthood [11].

rTMS protocol design elements that have been used to account for anatomical differences in youth are stimulation intensity adjustment and modeling of the TMS-induced electric field. rTMS stimulation intensity is tied to an individual’s resting motor threshold (RMT), which reflects the minimum intensity needed for a single pulse of TMS to the motor cortex (M1) to elicit an electromyographic (EMG) response in a target muscle. Higher stimulation intensity is needed to produce this motor response in children and adolescents vs. adults, likely due to immature corticospinal tract myelination and synaptic efficiency [16]. RMTs also have poorer reliability in youth and can be even higher in those with disorders linked to greater gamma-aminobutyric acid (GABA) concentrations in motor and sensorimotor cortices (e.g., ADHD; [17]).

On one hand, higher stimulation intensities raise concern for increased risk of adverse events (namely seizure induction), and some rTMS devices are limited in their ability to consistently deliver stimulation at very high intensities. On the other hand, the asynchronous maturation of motor cortex raises the possibility that RMTs in the *most mature* area of the cortex may result in under-dosing of rTMS when targeting the *least mature* cortical regions (e.g., prefrontal cortex) [13]. Current solutions emphasize de-risking protocols, such as delivering theta-bursting stimulation (TBS) at a lower frequency (e.g., 30 Hz instead of 50 Hz; [18]) or repeating RMT measurement longitudinally. Additional mechanistic testing, perhaps using computational or animal models, will be useful to refine parameters for non-motor cortical stimulation that optimally balance safety and efficacy. Following this with tolerability assessment in youth will be critical, as higher intensity stimulation, particularly in areas closer to the face and scalp nerves, can be intolerable.

Another solution for developmental tailoring is Finite Element Method (FEM) modeling [19]. FEM models segment tissue based on structural MRI images and assign tissue-specific electrical conductivities to estimate the propagation of the TMS-induced electric field at a specific cortical location. In this way, models account for anatomical differences that can meaningfully alter the field’s shape and distribution (e.g., skull thickness, coil-to-cortex distance, sulcal and gyral morphology). There is significant inter-individual variability in the TMS-induced electric field, and models in children suggest higher field strength, distribution, and within-group variability vs. adults [17]. Individual-person level FEM modeling is one solution that can be integrated in pediatric rTMS trials to enhance independent variable integrity (i.e., the likelihood that the induced electric field is maximally delivered to the area of interest; [20]).

FEM modeling across children, adults, and the elderly has shown higher peak fields in the white matter, gray matter, and cerebral spinal fluid in children, which decrease as a function of age [17]. The same study found that children exhibit thinner scalps and less coil-to-cortex distance than adults and the elderly, resulting in less current attenuation. Notably, anatomical differences that may be present in clinical disorders likely impact the induced electric field. For example, Mantell et al. [21] found that the presence of a brain lesion after perinatal stroke resulted in more variability in electric field strength. Research on rTMS for adolescent depression found that targets selected using FEM modeling resulted in greater electric field strength in the dorsolateral prefrontal cortex (DLPFC) compared to targets based on scalp measurements (5 cm rule, Beam F3) or anatomical MRI, and that median electric field magnitudes correlated with symptom improvement [22].

More work to address limitations in current FEM models is still needed. At present, modeling only represents the effect of a single TMS pulse and assumes high-quality structural MRI images (e.g., motion artifact, which is more common in youth, can confound tissue segmentation steps). These models can also be quite computationally demanding, limiting ultimate scalability to the clinic. Continued refinement of FEM modeling with developmental populations in mind will help enable accuracy and utility. An open question in this regard is whether and when it is appropriate to use normative or group-averaged head model datasets to inform targeting, rather than individual images [23]. Future research should include FEM modeling in large datasets of youth with and without neuropsychiatric illness to help us understand the tradeoffs between individual vs. group-based targeting and neuronavigation approaches.

While the optimal methods to account for neuroanatomical development in TMS trials are still unclear, neglecting to consider it could lead to misdoing of stimulation intensity: the distance between the TMS coil and cortical target, extracerebral tissue resistance, and cortical thickness and maturation status may differ significantly between the M1 target used for motor threshold determination and other common cortical targets, such as the dorsolateral prefrontal cortex (DLFPC). Over-dosing rTMS may lead to increased risk of adverse events such as seizure and recipient discomfort, while under-dosing rTMS could reduce treatment efficacy.

Future pre-clinical/animal model and human clinical research should focus on sharpening our understanding of how to consider typical and atypical cortical maturation timelines in rTMS protocol design. For example, is it better to stimulate cortical areas that have reached peak maturity, or to target these areas earlier? Are there harms to intervening during certain cortical developmental windows? Does the effect of particular rTMS stimulation parameters differ depending on the maturational stage of the tissue being stimulated? Does the timing of stimulation in relation to cortical development influence the trajectory of cortical maturation, or impact the durability and clinical efficacy of stimulation? Answering these questions will further our ability to identify how, when, and for whom rTMS may be a viable treatment option.

### Network topography

While rTMS protocols are often described in terms of the cortical ROI target (e.g., “left DLPFC stimulation”), we know that rTMS effects propagate across distributed intra-cortical and cortico-subcortical networks [24]. Because of this, rTMS efficacy can differ depending on network topography within the targeted ROI. For example, rTMS for TRD in adults has been shown to be most effective when delivered to a location within the DLPFC that has strong anti-correlation with the subgenual cingulate [25].

Cortical network topography undergoes considerable change across development. These changes are characterized by greater short-range functional connectivity earlier in childhood and increased long-range functional connectivity and network border refinement with age [26, 27]. Neurodevelopmental disorders [28], socio-economic factors [29], sex-related differences [30], and learning experiences [31] can further impact how network architecture unfolds over time. fMRI studies have revealed significant inter-individual heterogeneity in the size, shape, and spatial topography of these functional networks at all ages [32]. In children and adolescents, association networks show the most topographic variability [27]. Importantly, individual differences in network topography are related to individual differences in youth cognitive abilities [33] and psychiatric symptoms [34].

Consideration of the significant individual variability in network topography over development is important in rTMS trial design. For example, coil placements based on scalp heuristics or stereotaxic coordinates derived from group-average functional maps can result in stimulation of different functional networks across patients [35]. Overlooking this variability can threaten independent variable integrity and the reproducibility of a trial by increasing between-subjects variance in the network(s) being engaged by rTMS. This may produce results that obscure potentially promising network-based targets and cloud our ability to understand network-level mechanisms that underlie positive response.

The broader neuromodulation field is actively examining the extent to which TMS targeting should prioritize network-based targeting vs. scalp-, ROI-, or structurally-based targets, and whether clinical outcomes meaningfully differ when targeting is personalized and circuit-based. Literature addressing this question thus far has focused primarily on DLPFC stimulation for TRD in adults and results are mixed, though there is some suggestion that prospectively locating and personalizing connectivity-based targets may enhance clinical outcomes [36]. Because of the greater variability in network topography in youth, it will be important for future research to assess whether individual tailoring based on networks is related to clinical outcomes in developmental samples. Developmentally-focused clinical trials and TMS electric-field simulation studies will also be useful for understanding whether particular cortical regions (e.g., earlier maturing cortical regions) or rTMS protocols (e.g., H-coil, higher intensity stimulation) are more robust to heterogeneity.

Another open question is how to ensure we are accurately locating functional networks within individual children. Individually-based, network-level targeting in youth is currently possible (e.g., [37]) but its success depends on substantial computational resources and high-quality fMRI data (a potential barrier for youth who struggle with fMRI procedures). Methodological advances in fMRI image acquisition and data processing approaches to generate individual network topography maps (“precision mapping fMRI”; [38]) will help us better understand how to feasibly enact network-based targeting.

White matter tractography is another option for rTMS coil targeting in children and adolescents. Diffusion tensor imaging (DTI) work in adults has implicated that preferential sites of TMS activation lie in the sulcal wall rather than the gyral crown [39]. DTI has also been used to identify superficial nodes on the cortex that are reachable by TMS and have white matter connections to deep brain structures [40] implicated in mood disorders [41] and various neurological pathologies [42]. The combination of white matter tractography and electric field modeling may further optimize individualized targeting for enhanced circuit engagement for youth, but further evidence corroborating these approaches provide substantial benefit to treatment efficacy is needed to justify the computational burden.

Finally, because rTMS is inherently a network-targeting intervention, pediatric rTMS protocols should move beyond simply considering cortical ROIs that have “worked” in adults. It would be prudent to design protocols based on how rTMS might help engage aberrant and/or compensatory networks involved in specific domains of functioning. A major and pressing open question for the field to examine is whether, how, and for how long particular rTMS protocols actually impact network connectivity at particular stages of development, and how network-level changes relate to symptoms and clinical efficacy.

### Neuroplasticity

Durable after-effects of rTMS are thought to occur because of induced neuroplasticity. rTMS studies in primary motor cortex suggest that low- (1 Hz) and continuous theta-bursting frequencies of stimulation likely weaken synaptic strength via long-term depression (LTD)-like processes, while high- (5 Hz + ) and intermittent TBS likely produce sustained synaptic strengthening via long-term potentiation (LTP)-like effects [43]. However, the motor cortex is uniquely characterized by early maturation, large pyramidal neurons, and corticospinal axons with fast conduction time [44].

An important consideration is that the ultimate direction and durability of rTMS-induced neuroplasticity depends on the neuroplastic potential of the brain being stimulated (i.e., the principle of metaplasticity; [45]). Neuroplasticity is heightened in certain areas of the brain during critical windows of early childhood and adolescence, which may contribute to more rapid rTMS response. It may also complicate the cumulative effect of rTMS--for example, over-repetition of a particular rTMS sequence may interrupt or reverse the intended direction of modulation through homeostatic mechanisms [46]. Mechanistic, dose-response type-studies will be needed in the future to better understand the neuroplastic effects of different rTMS protocols in non-motor cortical areas at different stages of development (e.g., using tools such as TMS-EEG). Longitudinal assessment will inform our understanding of the durability of rTMS-induced changes. Though rTMS has been conventionally applied in adults with treatment-resistant psychiatric disorders, it is possible that heightened plasticity at younger ages will confer heightened rTMS efficacy and durability. Addressing these gaps will help us better understand “when, how, and how long” to intervene with rTMS.

### Neurotransmitters

The balance of excitatory (glutamate) and inhibitory (GABA) neurotransmitters (“E/I ratio”) changes as a function of age. The brain is excitation-dominant in early childhood due to an abundance of glutamatergic connections. Synaptic pruning and maturation of GABAergic circuits in mid-childhood create a more stable E/I balance [47] that is again imbalanced in adolescence, particularly in the prefrontal cortex, due to protracted development of local GABAergic networks [48]. E/I balance can be further altered in youth with specific disorders, such as autism, schizophrenia, ADHD, Tourette Syndrome, and depression.

rTMS can significantly change glutamate and GABA concentrations in the brain, although animal models suggest that specific effects vary by brain region [49]. Less is known about how developmental disruptions in E/I balance impact rTMS effects. Some research shows that GABAergic markers may be useful for predicting rTMS response. For example, youth with greater GABAergic system integrity in left DLPFC show greater response to DLPFC TBS [50, 51], and youth with ADHD and higher motor cortex GABA have higher RMTs and are less responsive to inhibitory TMS [16]. Potential concerns stemming from E/I imbalance include altered seizure risk, altered plasticity responses to particular rTMS protocols (e.g., excess glutamate can overactivate LTP-like processes), and, for high-frequency protocols, amplification of particular side effects (e.g., anxiety, irritability, manic-like symptoms). rTMS studies in youth that include longitudinal measurement of GABA and glutamate (e.g., with MR spectroscopy [16] or TMS and TMS-EEG indices of cortical inhibition [50]) will help inform how we consider E/I systems in the design of rTMS trials for youth.

### Hormones

Fluctuations in sex hormones can alter cortical excitability and synaptic plasticity [52]. Estrogen increases cortical excitability and neuronal plasticity by enhancing glutamatergic activity and reducing GABAergic neurotransmission, and progesterone reduces cortical excitability via GABAergic binding [52]. Testosterone has also been associated with higher cortical excitability, though in fewer studies [53]. The extent to which rTMS effects are moderated by endogenous hormonal variation across the menstrual cycle or by hormone-targeting treatments is incompletely understood, although there is evidence of impacts on RMT and behavioral outcomes [54]. Critically, no studies to date have examined how hormonal states interact with rTMS in relation to pubertal stage, irregular menstruation (common in adolescents), or gender-affirming hormonal treatments. Researchers have called for clinical trials evaluating the role of hormonal changes on rTMS-induced neural and clinical outcomes [54]. Including youth samples to answer these questions will be important. Approaches that will be useful to include in pediatric rTMS trials include assessment of pubertal state (e.g., Tanner staging), eligibility requirements for stability in hormone-targeting treatments prior to rTMS initiation, menstrual cycle tracking, and re-assessment of RMT across the menstrual cycle.

### State-dependent effects

It is well-established that rTMS’s immediate and long-term effects are dependent on behavioral activities and brain state around the time of stimulation at a scale of milliseconds to hours [55]. rTMS can be particularly impacted by states that are more dynamic or unstable in youth, including sleep patterns, nutrition/diet, level of wakefulness, substance intake (including caffeine), medication status, cognitive state (e.g., attention allocation, working memory, expectation, decision-making, content/valence of concurrent thoughts), and affective state (e.g., concurrent emotions, state and trait anxiety, reward and punishment sensitivity; [56]). Neuronal oscillations, which reflect transient local, network, and global brain states, can also modulate TMS effects, such as whether pulses are delivered at the peak, rising, trough, and falling phases of particular oscillatory bands [57]. For example, the same rTMS pulse sequence can induce LTP-, LTD-like, or no changes dependent on the timing of pulses in relation to an oscillation phase [58, 59].

There are a variety of strategies for managing the state-dependency of rTMS in pediatric trials. At a minimum, influential factors that can be tracked by youth and/or caregivers should be measured. For example, in our trials we administer daily forms to assess sleep, substance intake, and emotional state. This can be challenged by limitations of youth self-report (e.g., lower insight or metacognition, fear of disclosing illicit behavior) and parent-report (e.g., limited knowledge of child’s functioning when apart/at school), such that corroboration by multiple informants and objective tracking tools (e.g., smartwatch) may be useful. Approaches used to enable tolerability that can influence brain state should also be measured and explored as treatment moderators (e.g., music or movies for distraction).

State-dependency of rTMS can also be leveraged for treatment purposes. Research has shown synergistic effects of rTMS and simultaneous or sequential cognitive and behavioral interventions that co-engage neural circuits [60]. However, careful consideration about how to pair these interventions is warranted, as some combinations may be counter-productive [61]. Adjacent therapy to rTMS may be particularly critical for youth, who often lack the skills or the independent capacity to manage mental health symptoms effectively. Therapist-guided practice of behavioral and cognitive strategies during windows of TMS-induced neuroplasticity might potentiate outcomes and their durability. Pediatric studies testing combined rTMS and cognitive-behavioral therapies, such as ongoing trials [20, 37], will help inform our understanding of how interventions interact.

Finally, technological advancements to enable “brain state-dependent brain stimulation” may be useful in future pediatric studies. This approach focuses on individualizing the timing of TMS pulses, for example by using real-time EEG data to trigger TMS pulses according to the amplitude or phase of a certain EEG oscillation [57]. This method has shown initial clinical feasibility in adults with depression [62], but there may be added tolerability and technical challenges in youth (e.g., due to increased motion artifact, long EEG application time). Feasibility studies that focus on design elements to enhance tolerability in youth will be needed before this approach is scalable to larger clinical trials.

### Psychosocial development

Psychosocial development must always be considered in the design of any youth-focused intervention to enable feasibility and age-appropriate tailoring of procedures. For example, decisions about how to demonstrate or explain rTMS concepts, how to structure the physical space of the rTMS environment, how to consider factors that can influence brain state, and how much to involve parents/caregivers all require sensitivity to a youth’s psychosocial capacity.

Typical social-emotional development follows an expected trajectory, with milestones related to development of self (e.g., introspection, value identification), relationship to others (e.g., perspective taking, individuation from family, increased importance of peer/social relationships), and emotion/stress regulation (e.g., accurate threat appraisal, adaptive coping, cognitive reappraisal) [63]. Atypical neurodevelopment can perpetuate increased reliance on caretakers and decreased participation in social and school-based activities, causing delays in milestones related to social functioning and autonomy [64].

Adverse childhood experiences (ACES) and environmental unpredictability can have detrimental effects on neurodevelopment that may be relevant to consider in rTMS protocols. Evidence suggests accelerated neural development in trauma-exposed youth (i.e., reduced cortical thickness, increased white matter integrity; [65]), possibly via BDNF-mediated triggering of sensitive periods for neuroplasticity [66]. Timing of adversity can have region-specific effects, such that stressors in early childhood are more likely to impact subcortical networks and those in later childhood and adolescence to impact cortical regions, especially the PFC and those involved in reward processing (e.g., OFC, ACC; [67]). Particular types of adversity can also differentially affect the brain. For example, threat-related exposures most strongly influence regions in the amygdala-mPFC and salience networks, while deprivation more strongly affects frontoparietal network regions [68]. Trauma can also impact somatosensory and pain pathways, such that pain processing and somatic reactivity to uncomfortable stimuli, such as TMS pulses, may be altered [67]. It is important to underscore that there is heterogeneity in the brain’s response to adversity, and that adversity can also be followed by compensatory neuroplasticity processes that enable resilience [69]. Circuitry implicated in resilience include those subserving reward responses and cognitive control [70]--potentially interesting targets to include in future rTMS studies.

The potentially moderating role of trauma on rTMS outcomes has primarily been examined in adult studies of DLPFC stimulation for TRD. Results are mixed–some studies find greater likelihood of response amongst those trauma exposed [71], while others find lower response likelihood [72] or no relationship [73]. It is possible that trauma is more or less impactful for rTMS depending on its timing in relationship to neurodevelopment. It will be important for future research to assess the relationship between trauma/ACEs and rTMS effects in pediatric samples, and to consider how factors such as trauma timing and type may shape the function and maturational state of the tissue being stimulated. This work could help guide our understanding of whether rTMS procedures need to be adapted in the presence of a trauma history. It will also be useful to examine how rTMS may play a role in treating trauma-related disorders in youth, as well as explore whether it can help buffer against the adverse impacts of trauma on brain development.

To ensure rTMS trials appropriately account for participants’ expected psychosocial functioning, investigator teams should always include professionals with expertise in child development and experience working with the target population. Researchers should be aware of the range of psychosocial functioning in the target population and anticipate how it may intersect with capacity to assent/consent, feasibility (e.g., ability to adhere to logistics of study procedures and visit schedule), tolerability (e.g., capacity to cope with expected rTMS physical discomforts), and rTMS adjunctive therapies or supports. Patient-centered approaches that engage youth and caregivers as co-designers, consultants, or study advisory board members on rTMS trials (rather than only as participants) can help the research team proactively address potential barriers to study completion [74]. It will behoove the rTMS research community to encourage and value publication of protocols and qualitative studies that help facilitate sharing “lessons learned” about developmental/psychosocial tailoring in pediatric trials.

### Safety

The heightened plasticity and vulnerability of the developing brain calls for diligent safety management in the design and implementation of rTMS protocols, particularly in youth with neuropsychiatric disorders. All rTMS studies should follow best practice guidelines for TMS safety [75, 76] and practitioner training [77]. These include careful consideration of inclusion/exclusion criteria, medical screening and records review by a qualified member of the research team, and systematic collection of safety data at each study visit. For rTMS pediatric clinical trials, we recommend that the team include a physician or other medical professional with pediatric expertise to ensure developmentally sensitive evaluation of concurrent medications, medical comorbidities, and safety management.

A recent systematic review of safety data from 23 rTMS studies and 3 TBS studies in children with and without central nervous system disorders shows that most TMS-related adverse events (AEs) are mild and uncommon, with risk of any AE per session calculated at 0.0378 for rTMS and 0.1011 for TBS [78]. The most commonly experienced AEs are transient and mild and include headache, dizziness, muscle twitching, fatigue, general pain or discomfort, and nausea [78, 79]. More serious AEs have been reported but at a low frequency, including seizure (risk of 0.14% per session), neurocardiogenic syncope, and hypomania.

Although rTMS-induced seizures are rare, risk is higher in youth vs. adults given cortical hyper-excitability and higher RMT values. Visual observation of the participant during stimulation is required to monitor side effects in real time. The use of EMG to quantify motor-evoked potentials during RMT measurement (rather than relying on observation of visual twitch) avoids over-estimation of RMT, which may increase AE risk. EMG may also be used for physiological monitoring during rTMS to detect inadvertent spread of activation to motor regions [76]. Additional risk mitigation strategies include the use of active motor threshold (AMT) for dose titration (values are typically lower than RMT because spinal motor neurons are already active), use of biphasic pulses over monophasic in rTMS protocols [80], and incorporation of MRI-guided neuronavigation to improve RMT accuracy and reliable localization of the TMS coil across stimulation sessions. Another important consideration for youth is prevention of hearing damage, as young children have external ear canal resonance distinct from adults [81] and smaller head sizes, which results in less distance between the TMS coil and ears. TMS operators should model best practices for hearing protection and verify participants always wear hearing protection prior to discharging the TMS coil.

rTMS safety monitoring may be challenged by youth reluctance to accurately report relevant behaviors. For example, in a case report of a seizure during high-frequency rTMS in a 16-year-old female [82], post-seizure examination revealed the participant had an elevated blood alcohol concentration (0.20%) due to heavy drinking the evening prior that was not disclosed to study staff before rTMS delivery. Use of alcohol and other substances is not rare in youth–in 2023, 23% of high schoolers reported drinking alcohol in the past 30 days, followed by 17% reporting marijuana use [83]. Safety management can also be challenged by lifestyle factors that can impact cortical excitability and are common in youth, such as altered sleep patterns, poor diet, and inconsistent adherence to some types of medication (notably, these factors may also moderate rTMS’s clinical effects).

Valid reporting can be promoted by building trust with participants, including individual discussions with youth to explain the rationale behind questions that may feel intrusive (e.g., “this information helps us know if TMS is safe for you”) and to provide clarity about the bounds of confidentiality (i.e., what type of information does and does not need to be shared with parents). Asking substance use or mental health questions when a guardian is not present can facilitate accurate reporting. Mindful observation of the participant prior to rTMS administration also plays an important role in reducing risk (e.g., signs of intoxication, the smell of alcohol or smoke, fatigue, emotional distress, marked affect change, etc.). In our experience, we have found it valuable to document a participant’s baseline related to these factors and to ask about sleep duration, medication and substance use, and caffeine consumption in the past 24 h prior to each rTMS session. Future research should continue to examine how these variables relate to both the safety and efficacy of rTMS across development.

### AE monitoring

There is a critical need for systematic recording and reporting of AEs in pediatric rTMS studies. Mild to moderate AEs are often not included in publications [78]. In a systematic review of 56 pediatric neuromodulation studies, Lodewyk et al. [84] found that adverse event monitoring procedures were described in less than half of studies and that important intervention details were frequently absent, including stimulation parameters, exposure time (i.e., number and length of sessions), intervention setting, and training/qualifications of the TMS operators. The field also lacks a standard way of calculating AE “rates” (i.e., percentages are often reported without defining what the denominator represents). This highlights a need for improving the transparency and rigor of the methods used to monitor rTMS-related adverse events in youth, as well as for the field to set more rigorous expectations for AE-related reporting quality in trial pre-registrations and published studies.

Pediatric rTMS trials should collect AEs at each study visit using a structured questionnaire and grade responses for level of severity and rTMS relatedness. A comprehensive questionnaire was recently created to facilitate standardized collection and reporting of AEs in TMS studies [85] but may need developmental tailoring depending on the pediatric population of interest. Ultimately, AE measurement and determinations should include input from clinically-trained team members with expertise in the phenomenological characteristics of the target population. Efforts to update, revise, and disseminate AE questionnaires and monitoring procedures to fit different developmental cohorts should continue.

In our experience, developmental and psychiatric characteristics of youth participants can make AE evaluation challenging. These can include heightened somatic sensitivity, limited awareness of one’s own baseline symptomatology, and diminished metacognition and verbal abilities to describe internal experiences. Youth have high-rates of symptoms that can be common rTMS adverse events. For example, headaches, the most commonly reported rTMS AE, are more prevalent in youth vs. adults. Epidemiological studies suggest an overall prevalence of 54–62% for headache and 9–11% for migraine in children and adolescents [86, 87]. Migraines and chronic headaches also frequently co-occur with neurological and psychiatric disorders, including depression, anxiety, ADHD, and Tourette Syndrome [88]. For this reason, in our youth studies we document participants’ baseline experiences with headaches and other potential AEs in terms of typical phenomenology, intensity, frequency, course, functional impact, and typical treatments used. Including both youth and caregivers in a careful baseline assessment of AE-like symptoms is necessary for contextualizing possible rTMS-related changes. It would be beneficial for pediatric rTMS trials to report this baseline information alongside trial AEs. Future research investigating whether baseline status predicts experienced AEs may help highlight areas for risk mitigation or proactive tolerability management (e.g., prophylactic headache treatment, rTMS intensity titration).

### Tolerability

In clinical trials, “tolerability” is distinct from “safety.” “Safety” refers to medical risk to the participant while “tolerability” refers to the extent to which adverse effects can be tolerated by participants (i.e., their subjective experience of how it affects their quality of life; [89]). The most frequently used tolerability measure in pediatric TMS studies asks participants to rank the tolerability of TMS relative to 7 other common childhood experiences [90]. In a sample of n = 119 children receiving rTMS, first-session tolerability was ranked between “attending a birthday party” and “a long car ride” (and lower than “throwing up,” “going to the dentist,” and “getting a shot”), suggesting good tolerability [91]. Ratings were not correlated with age and improved across sessions. Continued efforts to use patient-centered tolerability measures will help inform decisions about efficacy-tolerability trade-offs.

Notably, Zewdie et al. [91] found that youth with depression, where frontal, high-frequency rTMS is applied, reported worse tolerability. Frontal targets near facial muscles, and anterior targets over facial nerves, may result in pain and discomfort. Youth with heightened sensory sensitivity and/or lower ability to tolerate discomfort/distress may have particular difficulty persisting with rTMS when this occurs. Adjustments to address tolerability in these cases can include lowering stimulation intensity, gradual ramp up of intensity, adjusting target location, and advance identification of alternate stimulation locations that reach the same circuit-based target. Procedural modifications to reduce the youth’s uncertainty can also help, including demonstrating the protocol (e.g., over a dummy head or stuffed animal), delivering a single pulse over the target area as an “example sensation,” and providing visual or verbal prompts that alert the child to stimulation onset. Notably, it may be difficult to assess stimulation-specific factors from non-specific procedural discomforts (e.g., neck pain from holding still). This highlights the importance of assessing tolerability in sham-controlled trials.

Overall tolerability may be promoted by environmental adaptations to the laboratory, including child-friendly room design, virtual pre-study equipment introductions for youth/families, individualized comfort measures guided by parent and child input, experimenter scripts with age-appropriate language and avoidance of potentially frightening wording (e.g., “the machine is overheating,” words like “zap” or “shock”). All participant-facing study staff should be trained on best practices when working with specific pediatric groups and disorder populations. In our experience, an often overlooked discipline that may be particularly helpful to include in rTMS trials is Child Life. Certified child life specialists are trained to develop developmentally-appropriate strategies to enable youth to participate in medical procedures, with a focus on reducing pain and fear, increasing participation, and reducing medical trauma [92]. The neuromodulation field should welcome qualitative and quantitative research focused on disseminating the efficacy of procedures that facilitate children’s ability to tolerate rTMS sessions.

### Feasibility

The social, environmental, and contextual factors in youths’ lives can impact rTMS feasibility and access to rTMS clinical trials. Currently approved FDA rTMS protocols for adults involve significant time commitment and transportation burden. The logistical, organizational, and financial demands of attending daily treatment sessions for weeks (or full-time treatment for a week, as in accelerated rTMS protocols) can be challenging for families–even more so for those who are low resourced, experiencing family-level dysfunction or distress, or encountering barriers to healthcare more broadly (e.g., due to geography, language, etc.). There are known patterns of ethnoracial disparities in adult rTMS clinical trials and in clinical access of neuromodulation treatments despite openness to try rTMS within racialized communities [93]. Even when families are able to access trials, they are often weighing participation against disruptions in school/work and extracurricular activities for the whole family system. Research teams should engage participant stakeholders at the stage of trial design to anticipate and problem-solve potential barriers to participation, ideally with an eye toward processes that will eventually be scalable to real-world clinical settings. It will also be helpful for rTMS trials to include frequent assessment of key outcome variables to enable generation of dose-response curves. Given heightened neuroplasticity in youth, it is possible that more brief courses of rTMS treatment, as compared to treatment-resistant adults, will yield meaningful clinical benefits. For example, fewer sessions or condensed protocols (e.g., accelerated TMS; [94, 95]) may be sufficient for some youth.

### Ethics

Ethical considerations for pediatric rTMS echo those of adult rTMS, with additional considerations given the heightened vulnerability of minors. As with all research in the United States, clinical trials using TMS must adhere to the ethical principles laid out in the Belmont Report, including respect for persons, beneficence, and justice [96].

### Respect for persons

Protection is required for individuals with diminished autonomy or capacity to consent, which includes minors. In rTMS trials including minors, a legal guardian acts as the consenting party. Institutional Review Boards (IRBs) or other regulatory agencies (e.g., US Food and Drug Administration for studies using rTMS as an investigational device) determine if the study population is capable of providing assent and whether it is required to be sought alongside consent of the guardian. The assent process provides minors the autonomy to decide if they are willing to participate or not (i.e., dissent) once they have an understanding of the research purpose and procedures while still acknowledging their diminished capacity to provide consent.

It can be challenging for children and parents to fully understand what rTMS procedures involve at the point of consent and assent. Efforts to enable autonomous decision making should include clear education about rTMS procedures and risks in developmentally-appropriate ways. For example, we use pictures and videos to help explain rTMS during consent, and we offer youth a pre-consent videochat to “see the machine” and ask staff questions. In addition to supporting autonomous decision making, this approach can also reduce anticipatory anxiety related to undergoing rTMS. Explaining risk in language that is easy to understand is also critical, such as avoiding overly-technical medical terminology and framing risk likelihood concretely (e.g., “For every 10 people who do TMS, 2 of them will feel a headache after.”). It can be helpful to reference tolerability studies comparing rTMS to typical childhood experiences [91]. Study team members obtaining assent should confirm understanding by having the minor describe study details back to them in their own words.

Researchers conducting rTMS trials in youth should also be mindful of factors that may unduly influence parental willingness to enroll a child in an rTMS trial. Caretakers who have exhausted other clinically available treatments for their child may be incentivized to pursue experimental interventions, or to pursue access to diagnostic assessments or therapies provided by clinical trials as a work-around for extended wait times to see a specialist in clinic. Clearly conveying the experimental nature of the rTMS being tested and confirming understanding can help avoid the possibility of therapeutic misconception (an inability to distinguish between the experiment methods of the clinical trials and an individualized treatment plan as one would receive in a medical setting). Contextualizing any experimental rTMS procedures within already available options for treatment can also help during consent.

### Beneficence

Clinical trials using rTMS must weigh the ratio of benefits to risks for children and adolescents (see “Safety” and “Tolerability” sections above). An important topic to highlight here is the idea of using rTMS for “neuroenhancement” to improve memory or executive function in otherwise neurotypical individuals. The ethics of this application require further ethical discussion, especially in adolescents and children [97]. While the neuroplasticity of the developing brain boasts potential for improved treatment efficacy for TMS, neuromodulation without a clinical basis may pose undue risk to youth during a dynamic period of neural organization, conceivably resulting in abnormal patterns of activity and emergence of pathophysiology. Policies must be created and enforced around neuromodulation for cognitive enhancement as these technologies become more readily available for off-label use.

### Justice

Inclusion of youth participants from a diverse array of backgrounds is needed, as there is a shortage of literature around racial and ethnic disparities in rTMS research. Inclusion of racial and ethnic minorities in pediatric TMS research samples may be hindered by: (1) a lack of diverse representation in research personnel, (2) distrust of human research for participants and their families grounded in historical abuse and injustices, and (3) exclusion of participants in clinical trials for cultural or phenotypic traits (e.g., hair texture) due to equipment limitations [98]. Simultaneously, the risks and benefits of pediatric rTMS research should be fairly distributed so that vulnerable populations do not undergo all the risk while more privileged ones benefit from medical discoveries and updates to clinical practice resulting from this work.

## Future research directions

With the FDA’s recent clearance of rTMS for adolescent depression, we are at an exciting point at which rTMS is now an “in-clinic” option for some youth receiving psychiatric care. Evidence for the safety, feasibility, and tolerability of rTMS in youth with a wide array of neurological and psychiatric conditions continues to grow, such that we are entering an era in which efficacy questions can become more central to pediatric rTMS studies. The appeal of rTMS is also clear–the ability to directly and non-invasively target the neural circuits driving symptoms allows for more precision in treatment targeting. It is a tool that may help translate psychiatric neuroscience findings into intervention. In our experience, parents and youth alike are also optimistic about rTMS, with some seeing it as an alternative to psychotropic medications, a potential “therapy booster,” or a line of hope when other treatments fail.

As we move into the “efficacy era” of pediatric rTMS research, it will be important for clinical trialists to approach this work with a deep appreciation and understanding of the nuanced ways that developmental factors can impact rTMS. Because rTMS is a brain-based intervention, it may be tempting to put all of our focus on biological mechanisms of development and how they interact with rTMS’s biological mechanisms of action. However, we must also give equal weight to the contextual and psychosocial aspects of development that influence whether, how, and how well rTMS will ultimately work for kids. For example, we know that MRIs “work” to image brain tissue, but successful acquisition of high-quality images in a 40-year-old vs. a 4-year-old involves very different procedural elements. As a field, we should expect, value, and invest in research that aims to develop procedures that enable youth with a range of ages, diagnoses, and cognitive/verbal abilities to participate in rTMS trials.

Trials should also be designed with clinical implementation in mind. Researchers should develop implementation-ready tools to enhance *fidelity*, such as written protocols, templates, or software tools to facilitate adherence to protocol parameters, delivery quality, and patient engagement; [99]. Principle or evidence-based guidelines for clinicians to follow when adjusting protocols for individual patients will also be needed (i.e., to enable *flexibility within fidelity* [100]. Implementation-focused research will also be needed to inform our understanding of how to best deliver rTMS to youth in the “real-world.” Pediatric-tailored healthcare often differs from the standard-of-care in adults in terms of provider type and team, setting, billing codes, safety management and reporting, and integration of family members into care plans. Evidence-based guidance about how to weigh and manage these factors in pediatric rTMS care will be valuable. We must also consider how best to support implementation of rTMS for youth in clinical care settings that are not pediatric-centric, such as clinics that serve patients across the lifespan or those in rural areas. As rTMS for adolescent TRD becomes more available, it will be extremely useful for clinicians and teams delivering this care to publish and share “lessons learned” that may help pave the way for efficient, effective dissemination of other rTMS protocols in the future.

It will also behoove the field to establish more clear standards for how pediatric rTMS studies should assess for and report adverse events and tolerability challenges. A standardized measure was recently developed for rTMS studies [101], but it was not designed with youth in mind. Developmentally tailoring tools like this will help enable more systematic reporting of mild to moderate adverse events and adjustments to address tolerability. Such details will help inform our understanding of potentially addressable moderators of rTMS discomfort. This information may also help shape the design of rTMS parameters and selection of rTMS treatment targets. For example, the left DLPFC and OFC are seemingly appealing targets because of efficacy in some adult conditions, but pain and discomfort in these frontal areas presents a significant tolerability barrier for many kids and teens. If we know particular regions/rTMS regimens are less tolerable, we can leverage our understanding of network topography and brain connectivity to potentially select more tolerable targets that engage similar circuitry.

Finally, as we extend rTMS into developmental samples, we should also re-consider the precedent set in adult research of focusing on treatment-resistant patients. This decision was made at a time when rTMS safety and efficacy was much less understood. Continuing to reserve rTMS for youth who are treatment-resistant would prevent us from examining rTMS’s strong potential to capitalize on neuroplasticity at early ages and stages of illness. Research should start asking questions about how rTMS might alter developmental trajectories toward health, *before* psychiatric illness de-rails youth and keeps them from having the experiences that shape healthy, productive adults.
